# Targeting the CXCR4/CXCL12 axis with the peptide antagonist E5 to inhibit breast tumor progression

**DOI:** 10.1038/sigtrans.2017.33

**Published:** 2017-08-11

**Authors:** Hua Guo, Yangyang Ge, Xiaojin Li, Yanlian Yang, Jie Meng, Jian Liu, Chen Wang, Haiyan Xu

**Affiliations:** 1 Department of Biomedical Engineering, Institute of Basic Medical Sciences, Chinese Academy of Medical Sciences & Peking Union Medical College, Beijing, People’s Republic of China; 2 CAS Center for Excellence in Nanoscience, National Center for Nanoscience and Technology, Beijing, People’s Republic of China; 3 University of Chinese Academy of Sciences, Beijing, People’s Republic of China

## Abstract

Emerging evidence has demonstrated that stromal cell-derived factor 1 (SDF-1) and its cognate receptor CXCR4 have critical roles in tumorigenesis, angiogenesis and metastasis. In this study, we demonstrated the significant inhibitory effects of a novel chemically synthetic peptide (E5) on the CXCR4/CXCL12 axis in breast cancer both *in vitro* and *in vivo*. E5 was capable of specifically binding to the murine breast cancer cell line 4T1, remarkably inhibiting CXCL12- or stromal cell (MS-5)-induced migration, and adhesion and sensitizing 4T1 cells to multiple chemotherapeutic drugs. Furthermore, E5 combined with either paclitaxel or cyclophosphamide significantly inhibited tumor growth in a breast cancer model. Mechanistic studies implied that E5 can inhibit the expression of CXCR4 to block the CXCL12-mediated recruitment of endothelial progenitor cells and repress CXCR4 downstream of the Akt and Erk signaling pathway, which are involved in tumor angiogenesis and progression. Further pharmacokinetic evaluation suggested that E5 has an acceptable stability, with a half-life of 10 h in healthy mice. In conclusion, E5 demonstrates a promising anti-tumor effect and could be a potential chemotherapeutic sensitizer to improve current clinical breast cancer therapies.

## Introduction

According to the latest cancer statistics, breast cancer has the highest incidence rates in women and is predicted to account for 30% of all new cancers among women in 2017.^1^ Although death caused by breast cancer has decreased in recent years, it was the leading cause of cancer death in women between 20 and 49 years of age prior to 2013, and remained that way in 2014 for women between 20 and 39 years of age.^1^ Therefore, there are still major challenges in breast cancer treatment, even though therapeutic reagents for increasing treatment efficacy have been intensively explored.^2^

Chemokine receptor 4 (CXCR4) is highly expressed in at least 23 various types of human cancers, including breast cancer, leukemia, melanoma, colorectal cancer, prostate cancer, ovarian cancer and lung cancer.^3^ When activated by its cognate ligand CXCL12, also called stromal-derived factor 1α (SDF-1α), which is secreted by stromal cells including fibroblasts and endothelial cells, CXCR4 mediates tumor cell survival and proliferation, which enhances primary tumor progression, angiogenesis and breast cancer metastasis.^4,5^ To date, a clear correlation between CXCR4 upregulation and tumor growth/progression, angiogenesis, invasion and migration has been well documented.^6,7,8^

Considering these factors, the CXCR4/CXCL12 axis is widely accepted as a potential therapeutic target for cancer therapy. Several promising CXCR4 antagonists are in various stages of development. Plerixafor (also termed as AMD3100), a CXCR4 commercial antagonist, has been approved by the FDA for hematopoietic stem cell mobilization as an injectable agent for short-term treatments,^9,10^ and it was able to block CXCR4 activation to inhibit metastasis of multiple solid tumors such as breast cancer,^11^ glioblastoma^12^ and melanoma.^13^ Moreover, AMD3100 increased the efficiency of multiple chemotherapeutics against leukemia, breast cancer, prostate cancer and glioma.^7,14^ Peptide T140 and its analogs blocking CXCR4 *in vitro* and *in vivo* have also been documented in numerous preclinical studies. *In vitro*, T140 effectively suppressed small cell lung cancer cell invasion into the extracellular matrix and adhesion to marrow stromal cells.^15^ TN14003 could inhibit the migration and invasion of pancreatic cancer cells caused by CXCL12 stimulator.^16^ T140 analog was able to overcome the protection of stromal cells to small cell lung cancer cells from apoptosis induced by an anticancer drug etoposide.^17^ *In vivo*, TN14300 or BKT140 could reduce intra-metastatic vascularization of pancreatic cancer cells and breast carcinoma, as well as head and neck tumor growth and metastasis.^16,18,19^ Peptide LY2510924 is a potent and selective CXCR4 antagonist for inhibiting renal cell carcinoma, lung, colon cancer and breast cancer that express functional CXCR4.^20^ Nevertheless, CXCR4 antagonists that get into clinical for treatment of solid tumors were rare at present.

We previously reported that a novel peptide, E5 (GGRSFFLLRRIQGCRFRNTVDD), interferes with the CXCR4/CXCL12 axis by significantly inhibiting stromal-induced activation in acute myelocytic leukemia cell lines *in vitro*,^21^ and enhancing the efficacy of chemotherapeutics in the acute myelocytic leukemia mouse model.^22^

Encouraged by the significant inhibitory effects of E5 on the CXCR4/CXCL12 axis, in this work, we applied E5 to breast cancer cells and mouse models to investigate whether E5 could sensitize breast cancer cells to chemotherapeutics and to reveal its underlying mechanisms. We showed that E5 improved the efficacy of multiple chemotherapeutics in mice bearing breast cancer tumors (4T1) by inhibiting tumor angiogenesis and tumor cell adhesion to stromal cells by blocking the CXCR4/CXCL12 axis.

## Materials and methods

### Cells and animals

The murine breast cancer cell line 4T1 and human umbilical vein endothelial cells (HUVECs) were purchased from the Cell Resource Center of the Chinese Academy of Medical Sciences (Beijing, China). The murine stromal cell line MS-5 was kindly gifted by Professor Bin Yin at the Cyrus Tang Hematology Center of Soochow University in China. Cells were cultured at 37 °C in a humidified incubator with 5% CO_2_ in RPMI 1640 or DMEM (Thermo Scientific HyClone, Logan, UT, USA) supplemented with 10% fetal bovine serum (Gibco, Grand Island, NY, USA), 100 U ml^−1^ penicillin and 100 U ml^−1^ streptomycin. The primary antibodies against cleaved caspase-3, phospho-Akt, Akt, phospho-Erk, Erk, β-actin were purchased from Cell Signaling Technology (Beverly, MA, USA), and anti-CXCR4 and anti-CD31 antibodies were purchased from Abcam (Cambridge, MA, USA).

Female BALB/c mice that were 5–6 weeks old were maintained at the Experimental Animal Center located at the Institute of Basic Medical Sciences at the Chinese Academy of Medical Sciences (Beijing, China) under specific pathogen-free conditions. The mice were fed autoclaved water and food pellets. All the animal experiments reported here were carried out in accordance with approved guidelines, and approved by the committee on the Animal Care and Use of the Institute of Basic Medical Sciences at the Chinese Academy of Medical Sciences & Peking Union Medical College.

### E5 peptide

The E5 peptide studied here was obtained by cell-based selection as described previously and purchased from GL Biochem (Shanghai, China).^21,22^

### Flow cytometry

The expression of the cell surface CXCR4 protein and the affinity of E5 for 4T1 cells, MS-5 cells and HUVECs were analyzed by the Accuri C6 flow cytometer (BD Biosciences, San Jose, CA, USA). Briefly, 1×10^5^ cells were seeded in 24-well culture plates, incubated overnight for adhesion and collected with 0.5 mM EDTA solution. After centrifugation, cells were incubated with Rabbit anti-CXCR4 polyclonal antibody (1:100) in phosphate-buffered solution (PBS) for 40 min at room temperature, followed by incubation with a secondary DyLight 649 Donkey anti-rabbit IgG antibody (BioLegend, San Diego, CA, USA) for 30 min at 4 °C. For the affinity assay, collected cells were incubated with 0.1 mM biotin-E5 for 40 min at room temperature and then stained with fluorescein isothiocyanate (FITC) Streptavidin (BioLegend) for 30 min at 4 °C. After a final wash, the sample cells were subjected to flow cytometry. A total of 1×10^4^ cells were collected, and acquired data were analyzed by CFlow Plus software (Accuri Cytometers, Ann Arbor, MI, USA).

### Cell proliferation and apoptosis assay

The effect of E5 on the viability of 4T1 cells was determined using Cell Count Kit 8 (CCK-8, Dojindo Molecular Technologies, Kumamoto, Japan) according to the manufacturer’s protocol. Briefly, 8×10^3^ cells were seeded in 96-well culture plates and incubated overnight. Then, the cells were treated with different concentrations of E5 (1–100 μM) for 24 h at 37 °C. After being washed with PBS twice, 10 μl of the CCK-8 reagent (in 100 μl of medium) was added, and the mixture was incubated for an additional 2 h. The absorbance was measured at 450 nm using the Synergy H1 Hybrid Multi-Mode microplate reader (BioTek Instruments, Winooski, VT, USA). Wells containing only medium (no cells) served as blank controls. The absorbance value of cells in the absence of E5 was set as 100%.

4T1 cell apoptosis was quantified using the Annexin V/Propidium Iodide (PI) apoptosis kit (eBioscience, Vienna, Austria) according to the manufacturer’s protocol. Briefly, 1×10^5^ cells were seeded in 24-well culture plates and incubated overnight. After being treated with E5 (1–100 μM) for 24 h, the cells were collected and resuspended in binding buffer. Then, 5 μl of FITC-Annexin V was added for 3 min, followed by the addition of 10 μl of PI for 10 min. The resulting cells were subjected to flow cytometry.

### Western blot

After being treated with different concentrations of E5 (1–100 μM) for 24 h, cells were collected and subjected to caspase-3 signaling analysis. The western blot was performed as described previously.^21^ Briefly, cells were lysed in RIPA lysis buffer with protease and phosphatase inhibitors at 4 °C for 30 min. The lysates were centrifuged for 15 min at 12 000 r.p.m. The proteins were loaded and resolved on 12% polyacrylamide gels (Applygen, Beijing, China) and electroblotted to PVDF membranes (0.45 μm, Millipore, Bedford, MA, USA). The blots were blocked with 5% non-fat milk in Tris-buffered saline containing 0.1% Tween-20 (TBST) for 2 h and probed with anti-cleaved caspase-3 antibody in 5% non-fat milk in TBST overnight at 4 °C. After being washed with TBST, the blots were incubated with the corresponding secondary antibodies. After a final wash, the antibody-antigen complexes on the blots were detected using Image Quant LAS 4000 (GE Healthcare Life Science, Pittsburgh, PA, USA).

### Transwell assay

All cell migration assays were conducted in a Boyden chamber modified such that Transwell inserts (pore size=5 μm, Millipore, Zurich, Switzerland) were placed in 24-well plates. To assess the migration mediated by CXCL12 (R&D Systems, Minneapolis, MN, USA), 4T1 cells or HUVECs were pre-treated with different concentrations of E5 (0.1–10 μM) in serum-free medium (opti-MEM, Life Technologies, Grand Island, NY, USA) for 1 h at 37 °C, and 8×10^4^ cells from each cell line were then added into the upper chambers of the inserts. The lower chambers were filled with 800 μl of culture medium with or without CXCL12 (200 ng ml^−1^). To examine the migration mediated by conditional medium from stromal cells that secrete CXCL12, 1×10^5^ MS-5 cells were pre-seeded in 24-well culture plates with 800 μl complete medium and incubated for 48 h to allow for mass secretion of CXCL12. Then, the medium was collected and added to the lower chamber. Next, 8×10^4^ 4T1 cells pre-treated with E5 (10 μM) in serum-free medium for 1 h at 37 °C were seeded in the upper chambers. After a migration time of 24 h, the cells in the insert were removed by wiping with a cotton swab, and cells adhering to the bottom of the insert were fixed with 4% paraformaldehyde/PBS for 10 min. Then, the inserts were washed with PBS followed by staining with 0.1% crystal violet (HBK Pharmaceutical Technology, Beijing, China) for 1 h. After being rinsed with PBS, the crystal violet was eluted by decoloring solution. Migrating cells were estimated by measuring the OD_570 nm_ with the Synergy H1 Hybrid Multi-Mode microplate reader (BioTek Instruments).

### Adhesion assay

Briefly, 1×10^4^ MS-5 cells were pre-seeded in 96-well culture plates and incubated for 48 h to allow for the mass secretion of CXCL12. Then, 4×10^4^ 4T1 cells pre-treated with different concentrations of E5 (0.1–10 μM) for 2 h in serum-free medium were added, and the mixture was incubated for an additional 2 h. Non-adherent cells were gently removed from the wells, and the number of adherent cells was measured with the CCK-8 kit. The absorbance value of the 4T1 cells in the absence of E5 was set as 100%.

### Drug sensitivity in the co-culture system

Briefly, 2×10^4^ MS-5 cells were seeded in 96-well culture plates and incubated overnight. 4T1 cells pre-treated with E5 (10 μM) for 2 h in serum-free medium were added to the plates with or without MS-5 cell layers, and the mixture was incubated for an additional 4 h prior to being treated with chemotherapeutic drugs (paclitaxel (10 μM), elemene (10 mg l^−1^), and cisplatin (8 μM), acquired from Peking Union Medical College Hospital, Beijing, China) for 24 h. The viability of the 4T1 cells was analyzed by the CCK-8 assay. The absorbance value of the 4T1 cells in the absence of drug treatment was set as 100%.

### Drug sensitivity under CXCL12 stimulus or conditional medium

Briefly, 1×10^4^ 4T1 cells were pre-seeded in 96-well culture plates and incubated overnight. Then, the cells were treated with 10 μM E5 in serum-free medium for 2 h, followed by the addition of either complete medium with 200 ng ml^−1^ CXCL12 or conditional medium obtained from 48 h of incubation with MS-5 cells for an additional 4 h of treatment. After that, the cells were incubated the chemotherapeutic drugs paclitaxel (4, 20 and 100 nM), elemene (10, 20 and 40 mg l^−1^), or cisplatin (2, 4 and 8 μM) for an additional 24 h. The 4T1 cell viability was analyzed using the CCK-8 assay. The absorbance value of 4T1 cells in the absence of drug treatment was set as 100%.

### Animal experiment

For animal experiments, 1×10^6^ 4T1 cells were orthotopically inoculated into the right fourth mammary fat pat of BALB/c mice. Seven days after inoculation, the mice were randomized in six groups (*n*=9 for each group): the control group, the E5 (40 mg kg^−1^) group, the chemotherapeutic drug groups (cyclophosphamide (CTX, 90 mg kg^−1^) or paclitaxel (PTX, 8 mg kg^−1^)), and a group consisting of E5 combined with one of the drugs. E5 solution was subcutaneously injected around solid tumors every other day, and drugs were intraperitoneally injected once a week. The control tumor-bearing mice were treated an equal volume of solvent. The tumor sizes were measured every four days with a caliper. Tumor volume was calculated according to the formula: *V*=(*a*×*b*^2^)/2, where *a* and *b* are the maximal and minimal diameter in millimeters, respectively. On day 33 after cell injection, the mice were killed by cervical dislocation. The tumors and main organs from each group were weighed immediately after dissection and preserved at −80 °C for further analysis.

### Tumor lysates

Frozen-tumor tissues <1 cm thick were crushed with a precooled tissue bead mill homogenizer and lysed in RAPI buffer with protease and phosphatase inhibitors. Total protein samples (40 μg) were loaded, and western blot was performed as described above. The primary antibodies used were anti-phospho-Akt, anti-Akt, anti-phospho-Erk, anti-Erk, anti-β-actin, anti-CXCR4 and anti-CD31.

### *In vivo* pharmacokinetics of E5

Pharmacokinetic studies of E5 were performed in healthy female BALB/c mice. E5 conjugated to FITC (FITC-E5) was subcutaneously injected into mice at different time points (40 mg kg^−1^, *n*=3 for each group). The free FITC of the same concentration to FITC-E5 was set as a control (*n*=3). Then, the blood samples were collected and centrifuged at 3000 r.p.m. at 4 °C for 10 min, and the fluorescence of the serum samples was measured using the Synergy H1 Hybrid Multi-Mode microplate reader (BioTek Instruments) at an excitation wavelength of 488 nm and an emission wavelength of 525 nm. Meanwhile, the mice were killed, and the liver and kidney were collected for fluorescence imaging using a Xenogen IVIS Spectrum system (Caliper Life Science, Hopkinton, MA, USA).

### Statistical analysis

All experiments were carried out at least three times, and Student’s *t*-tests were performed to assess the statistical significance of the results (*P<0.05 and **P<0.01).

## Results

### E5 has specific affinity to 4T1 breast cancer cells and HUVECs

**Figure 1:**
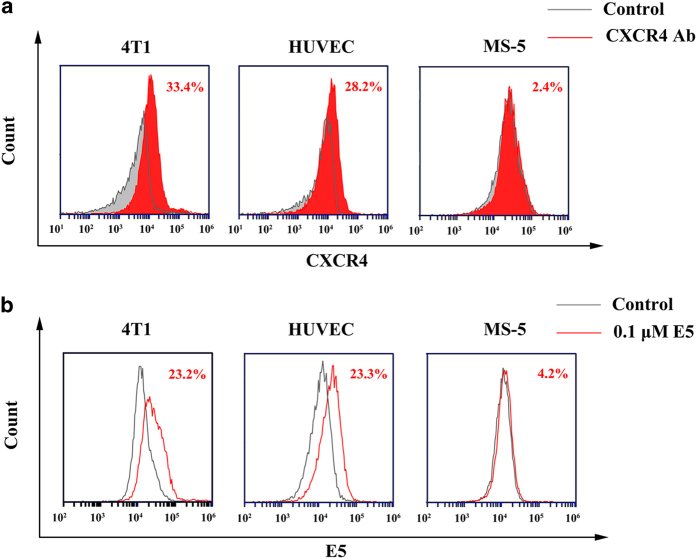
Affinity of E5 to 4T1 cells, human umbilical vein endothelial cells (HUVECs) and MS-5 cells. (**a**) CXCR4 expression determined by flow cytometry using a CXCR4 antibody. (**b**) Affinity of E5 (0.1 μM) to 4T1 cells, HUVECs and MS-5 cells.

First, we examined the expression of CXCR4 in a murine breast cancer cell line (4T1), HUVECs and a murine stromal cell line (MS-5). Flow cytometry results showed that the 4T1 cells and HUVECs had high levels of CXCR4 expression, whereas MS-5 cells expressed low level of CXCR4. The CXCR4 levels in 4T1 cells, HUVECs and MS-5 cells were 33.4%, 28.2% and 2.4%, respectively (Figure 1a). Meanwhile, E5 (0.1 μM) had high affinity towards 4T1 cells and HUVECs (Figure 1b), as the percentages of fluorescent cells were 23.2% and 23.3%, respectively. In contrast, E5 had low binding affinity towards MS-5 cells, as the E5 positive rate was 4.2%. These results suggest that E5 has specific affinity to cells that highly express CXCR4.

### E5 is cytotoxic to breast cancer cells in a concentration-dependent manner

**Figure 2:**
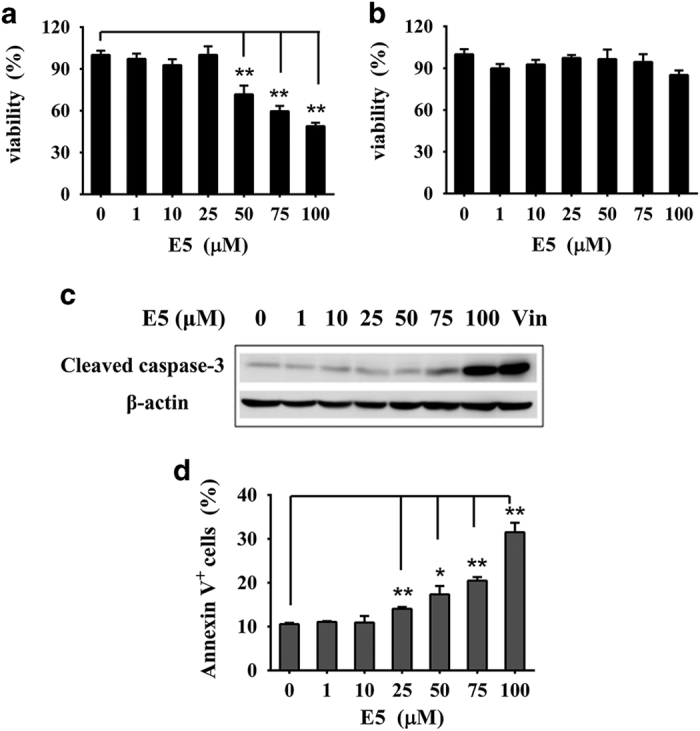
The cytotoxicity of E5 to 4T1 cells and human umbilical vein endothelial cells (HUVECs). Cells were treated with E5 at different concentrations for 24 h. The viability of 4T1 cells (**a**) and HUVECs (**b**) was measured with the CCK-8 assay. (**c**) The activation of caspase-3 signaling induced by E5 in 4T1 cells. A total of 3 nM vincristine was used as a positive control. (**d**) The apoptosis levels of cells measured by FITC-Annexin V/PI double staining. The data are presented as the means±s.d. (*n*=3). * represents a significant difference between the experimental groups and the untreated group (*P<0.05 and **P<0.01).

To examine the cytotoxic effect of E5, the CCK-8 assay was conducted with 4T1 cells and HUVECs exposed to E5 for 24 h, with concentrations ranging from 1 to 100 μM. As shown in Figure 2a, when the E5 concentration was <25 μM, the viability of 4T1 cells was >90%, which proves that E5 is non-toxic to 4T1 cells at this concentration. However, as the concentration was increased from 50 to 100 μM, the cell viability decreased from 72 to 49%, suggesting that E5 is cytotoxic to 4T1 cells at high concentrations. However, E5 did not show obvious cytotoxicity to HUVECs at concentrations up to 100 μM (Figure 2b). The apoptosis assay was performed to confirm cell death using Annexin V-FITC/PI double staining. As expected, E5 induced 4T1 apoptosis in a concentration-dependent manner (Figure 2d). The percentage of apoptotic cells increased from 14 to 32% as the E5 concentration was increased from 25 to 100 μM, which is consistent with the CCK-8 assay results. As the E5 concentration increased, E5 clearly induced the activation of apoptotic signaling at concentrations of 75 and 100 μM, as evidenced by the western blot assessing cleaved caspase-3 (Figure 2c). Vincristine, a conventional chemotherapeutic drug that can induce the activation of apoptosis through caspase-3 signaling, was set as a positive control. At 100 μM, E5 has an equivalent effect as that of vincristine at 3 nM. Considering its cytotoxicity, we used E5 concentrations <25 μM for the remaining *in vitro* experiments.

### E5 inhibits the migration of 4T1 cells or HUVECs mediated by CXCL12 and inhibits the adhesion of 4T1 cells to stromal cells

**Figure 3:**
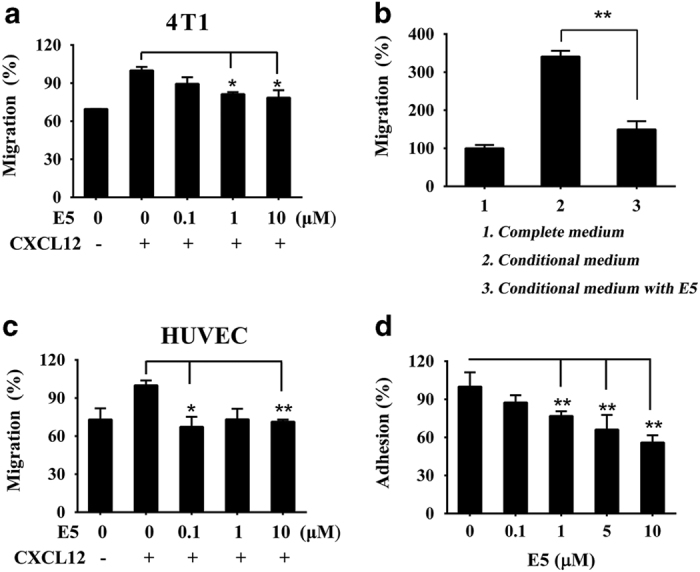
E5 inhibited the migration of 4T1 cells or human umbilical vein endothelial cells (HUVECs) induced by CXCL12 and the adhesion of 4T1 cells to MS-5 cells. 4T1 cells (**a**) or HUVECs (**c**) in the upper chamber were treated with E5 at different concentrations, and the lower chamber contained 200 ng ml^−1^ of CXCL12. (**b**) 4T1 cells pre-treated with E5 (10 μM) were seeded in the upper chamber, and the lower chamber was filled with the conditional medium prepared from the MS-5 incubation. (**d**) MS-5 cells were pre-incubated for 48 h, followed by the addition of 4T1 cells pre-treated with E5 at different concentrations. The CCK-8 assay was applied to calculate the adhesive rate by detecting the cell viability. The data are presented as the means±s.d. (*n*=4). * represents *P*<0.05 and ** represents *P*<0.01, respectively.

Studies on the CXCR4/CXCL12 axis have shown that CXCL12 is highly secreted by regional lymph nodes, lung, liver and marrow, and that CXCL12 can stimulate CXCR4-expressing tumor cell motility and invasiveness.^14^ Meanwhile, CXCL12 in tumor microenvironments can also induce the recruitment of endothelial progenitors for tumor angiogenesis.^23^ To determine the effect of E5 on the chemotaxis of 4T1 cells in response to CXCL12 and MS-5 conditional medium, the transwell assay was performed. The supplement of CXCL12 in the lower chamber strongly enhanced the migration rate of both 4T1 cells and HUVECs compared with that of the random migration group (Figure 3), and E5 could inhibit the migration activity of 4T1 cells and HUVECs induced by CXCL12 in a concentration-dependent manner. The cell migration percentages were 89±5.2%, 81±1.6% and 79±5.8% for 4T1 cells (Figure 3a) and 72±7.5%, 81±3% and 79±0.9% for HUVECs (Figure 3c) when cells were pre-treated with E5 at concentrations of 0.1, 1 or 10 μM, respectively. Similar to CXCL12-induced migration, the migration index of 4T1 cells exposed to MS-5 conditional medium containing CXCL12 was also markedly increased compared with that of cells exposed to the normal medium alone (set as 100%). When E5 (10 μM) was introduced, it significantly inhibited the migration rate, which decreased from 340.9 to 149.7% (Figure 3b). The MS-5 conditional medium used here capably provided an effective concentration of CXCL12, as measured by the ELISA assay (Supplementary Figure S1). Taking these results together, we demonstrated that E5 interrupted the chemotactic response of 4T1 cells to both CXCL12 and MS-5. In addition, the response to CXCL12 activation on the recruitment of endothelial cells was also inhibited.

CXCR4/CXCL12 can mediate the adhesion of tumor cells to stromal cells in tumor microenvironments, and the resulting interactions provide survival, anti-apoptosis and drug-resistance signals.^24,25^ Therefore, we next studied whether E5 could inhibit the adhesion of 4T1 cells to MS-5 cells. As shown in Figure 3d, E5 significantly decreased the population of 4T1 cells that adhered to MS-5 cells in a concentration-dependent manner, indicating that E5 may also disrupt adhesion by interrupting the CXCR4/CXCL12 axis. In our previous work, it has been demonstrated that E5 inhibited cell adhesion by disabling the skeleton organization as well as downregulated CXCL12-induced Akt, Erk and p38 phosphorylation.^21^

### E5 enhanced the sensitivity of 4T1 cells to chemotherapeutics

**Figure 4:**
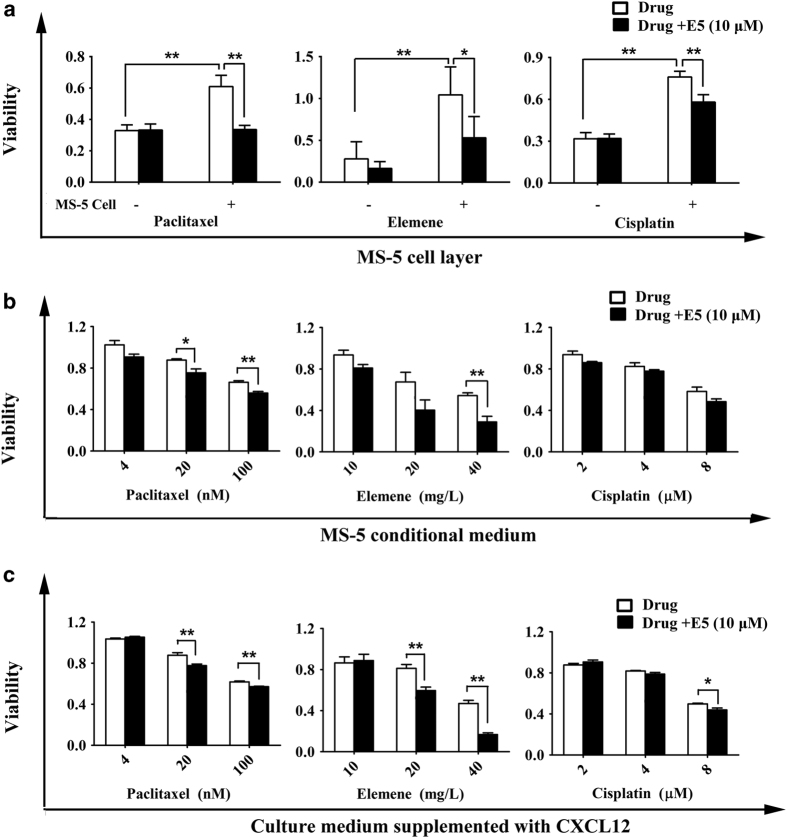
E5 enhanced the sensitivity of 4T1 cells to different chemotherapeutic drugs in co-culture models. (**a**) The cytotoxicity of paclitaxel (10 μM), elemene (10 mg l^−1^), and cisplatin (8 μM) on 4T1 cells pre-treated with E5 (10 μM) and co-cultured with MS-5 cells. The viability of 4T1 cells pre-treated with 10 μM E5 with paclitaxel (4, 20 and 100 nM), elemene (10, 20 and 40 mg l^−1^) or cisplatin (2, 4 and 8 μM) for 24 h in the presence of MS-5 conditional medium (**b**) or medium supplemented with CXCL12 (**c**). The viability percentage of 4T1 cells in the absence of treatment was set as 100%. The data are presented as the means±s.d. (*n*=4). * represents *P*<0.05 and ** represents *P*<0.01, respectively.

To investigate whether E5 could overcome the tumor microenvironment-induced drug resistance and increase the sensitivity of 4T1 cells to chemotherapeutic drugs, we combined E5 with multiple drugs (paclitaxel, elemene and cisplatin) in a co-culture system. The 4T1 cells were pre-treated with 10 μM E5 and then co-cultured with or without MS-5 cells, MS-5 conditional medium or medium containing 200 ng ml^−1^ CXCL12 in the presence of paclitaxel, elemene or cisplatin. The results showed that MS-5 cells could protect 4T1 cells from drug-induced death and increase the viability of cells compared to cells without the MS-5 cell layer (Figure 4a). Moreover, E5 could partly reverse the protection provided by MS-5 cells and sensitize 4T1 cells to the chemotherapeutic drugs. However, in the absence of MS-5, E5 did not show this effect (Figure 4a). Similarly, in the presence of MS-5 conditional medium or the medium supplemented with CXCL12, E5 could enhance the cytotoxic effects of paclitaxel (4, 20 and 100 nM), elemene (10, 20 and 40 mg l^−1^), and cisplatin (2, 4 and 8 μM) to 4T1 (Figures 4b and c).

### E5 in combination with chemotherapeutic drugs inhibited tumor growth in a breast cancer model

**Figure 5:**
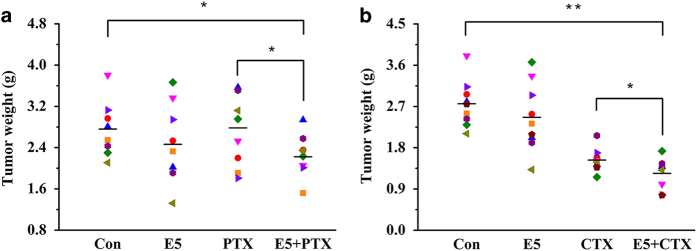
The tumor masses in the different groups (*n*=9). BALB/c mice were subcutaneously inoculated with 4T1 cells. After 7 days, the mice in each group were subcutaneously injected with E5 every other day and intraperitoneally treated with paclitaxel (PTX) (**a**) or cyclophosphamide (CTX) (**b**) once a week. * represents *P*<0.05. ** represents *P*<0.01.

To evaluate the *in vivo* efficacy of E5 combined with chemotherapeutic drugs in a breast cancer model, we established a tumor xenograft model in BALB/c mice by subcutaneously inoculating 4T1 cells into the right fourth mammary fat pat. Mice were administered 40 mg kg^−1^ E5 subcutaneously every other day and 8 mg kg^−1^ paclitaxel (PTX) or 90 mg kg^−1^ cyclophosphamide (CTX) once a week intraperitoneally. After 33 days of tumor inoculation, excised tumors from each group were weighed. In the current study, a low dose of PTX was employed, aiming to highlight the sensitizing effects of E5. It was shown that PTX or E5 alone at the dose used in this study did not show a significant inhibitory effect on the tumor weight compared with the control group. When E5 was administrated together with PTX, the combination demonstrated a significant inhibition of tumor growth compared with control group (Figure 5a). The clear anti-tumor effect using E5 together with a low dose of PTX may pose a promising strategy to reduce the side effects associated with the high dosage of PTX chemotherapy *in vivo*. The similar pattern of E5 facilitated the anti-tumor effect was observed in combination with CTX. As shown in Figure 5b, the tumor burden in the E5 with CTX group was reduced compared to the CTX alone group, which were consistent with the results obtained from tumor growth curve (Supplementary Figure S2a). The results showed that the tumor burden of the E5 plus PTX (Figure 5a) or CTX (Figure 5b) group was reduced compared to that of the PTX or CTX groups alone, which is consistent with the results obtained from the tumor growth curve (Supplementary Figure S2a). In addition, E5 alone had marginal anti-tumor effects. Specifically, we observed that the mice in the E5+CTX group, with sleek fur, were in better condition than the mice in the CTX group, which had ruffled fur, implying that the latter group was possibly suffering more from the disease (Supplementary Figure S2c). Most importantly, a significant prolonged survival was observed in the E5+CTX group compared with that of the CTX group (Supplementary Figure S2b). Collectively, E5 could be a general sensitizer for the cytotoxic cancer drug as revealed by PTX and CTX whose pharmacologic mechanisms were distinctive.

**Figure 6:**
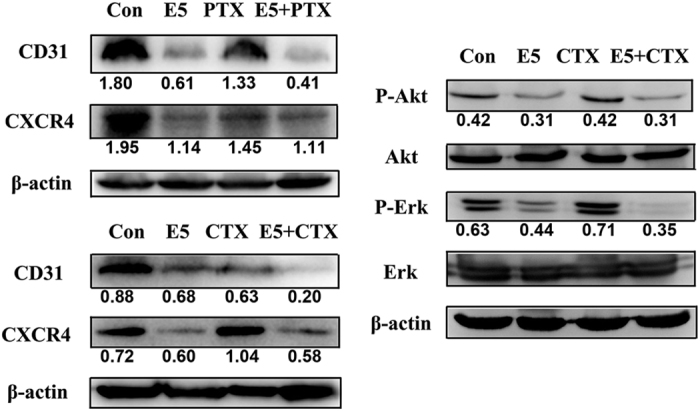
Western blot analysis of CD31, CXCR4, and Akt and Erk phosphorylation in tumors of mice treated with either E5 or E5 combined with paclitaxel (PTX) or cyclophosphamide (CTX).

To understand the molecular mechanism of tumor growth inhibition by E5 in this model, excised tumor tissues were collected and assessed with western blot analysis. CD31 (endothelial cell marker) and CXCR4 expression, as well as Akt and Erk phosphorylation, were evaluated. As shown in Figure 6, the E5, the E5+PTX group and the E5+CTX group had significantly decreased levels of CD31, indicating the recruitment of endothelial progenitors for tumor angiogenesis was suppressed by E5. Meanwhile, the downregulation of CXCR4 induced by E5+PTX and E5+CTX clearly showed the antagonistic effects of E5. Regarding protein signaling, the E5 and the E5+CTX groups significantly inhibited the levels of Akt and Erk phosphorylation compared to those of the control group (Figure 6). These results indicate that E5 enhances the efficacy of chemotherapeutic drugs by downregulating the phosphorylation of the signaling proteins Akt and Erk, which is a result of the antagonistic effects of E5 on the CXCR4/CXCL12 axis.

### E5 had good *in vivo* stability through hepatic metabolic clearance

**Figure 7:**
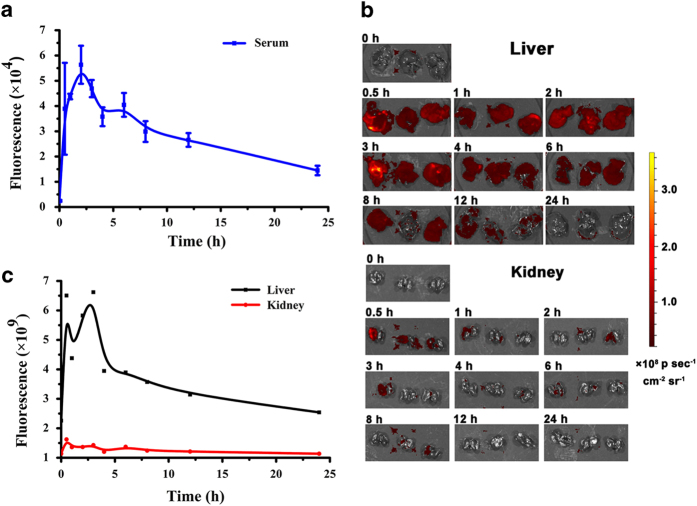
Pharmacokinetic profile of E5 subcutaneously administered to mice at different time points (*n*=3). The E5 concentration in circulating blood and main organs was measured with fluorescence imaging and quantified using FITC-conjugated E5. (**a**) The fluorescence intensity in the serum samples after FITC-E5 injection. (**b**) The fluorescence images of livers and kidneys at different time points. (**c**) The liver and kidney fluorescence intensity curve.

To assess E5 metabolism, pharmacokinetic experiments were performed by subcutaneously injecting FITC-conjugated E5 into healthy BALB/c mice. After administration at different time points, samples of mice serum were collected, and the fluorescence versus time profiles are shown in Figure 7a. The concentration of E5 peaked in the blood 2 h after subcutaneous administration and gradually decreased within 24 h. On the basis of the curve, E5 demonstrated good stability *in vivo*, and the mouse half-life was ~10 h. It was also observed that the fluorescence intensity in the mouse liver was much higher than that in the kidney (Figure 7b), and the fluorescence versus time profiles in the liver were similar to those in the circulating blood (Figure 7c). These observations suggest that E5 delivered subcutaneously was mainly cleared through the hepatic metabolic pathway. Meanwhile, pharmacokinetic of free FITC as a control in healthy mice was also evaluated. As shown in Supplementary Figure S3, free FITC by subcutaneous administration was cleared both through hepatic and kidney metabolic pathway, which is different from FITC-E5.

## Discussion

Breast cancer is one of the most common cancers affecting females worldwide and has continuously increasing incidence and mortality rates. Thus, studies of new therapeutic strategies are still important and demanded. The CXCR4/CXCL12 receptor/ligand pair has been shown to have a critical role in the progression of many kinds of cancers, including lung cancer,^26^ colon cancer,^27^ ovarian cancer,^28^ melanoma,^29^ leukemia ^30^ and breast cancer. More importantly, CXCR4 expression is a prognostic marker in breast carcinoma.^31^

To present, various antagonists targeting CXCR4 have been developed for uses in a variety of preclinical tumor models. Nevertheless, challenges still exist in clinical applications. AMD3100 is reported to display a weak partial agonist activity.^32^ Peptide antagonist T140 and its analogs are derived from the horseshoe crab and have a short *in vivo* half-life. Contrary to these, E5 is artificially designed and chemically synthesized, which makes its features easy and economically viable to prepare, avoiding the potential risks of biological substances. In addition, it is known that existing peptide antagonists generally have a short *in vivo* half-life. For example, the half-life of BKT140 and CTCE-9908 is <1 h;^33,34^ the promising peptide LY2510924 has a half-life of 3–5 h in preclinical species.^20^ Strikingly, E5 has a much longer half-life of 10 h, suggesting a relatively high stability *in vivo* and the potency of less administration for cancer treatment. Most importantly, versatility of E5 to sensitize different chemotherapeutics has been demonstrated in both *in vitro* and *in vivo* models.

The cytotoxicity of blocking CXCR4 with antagonists depends on the concentration and the cell types. For instance, CXCR4 antagonist BKT140 could induce 15–35% cell death at 4 or 8 μM. When its concentration was high as 20 μM, the cell death rate was about 60%.^35^ In addition, BKT140 displayed selective toxicity toward acute myelocytic leukemia and multiple myeloma cells, inducing cell apoptosis or death of different degree.^36^ As for E5, it did not induce cell death directly below 30 μM in multiple acute myeloid leukemia cell lines, whereas induced mild apoptosis in HL60, U937 and THP-1 (not higher than 25%) when the concentration was ranged from 30 to 80 μM, but induced 60% apoptosis of NB4 cells when E5 concentration reached 80 μM.^21^ In the current study, E5 induced certain degree cell apoptosis (about 14–32%) when the concentration was ranged from 25 to 100 μM. In many cases including our research, the most expected effect of CXCR4 antagonist is to increase the sensitivity of tumor cells to therapeutics by blocking CXCR4, instead of killing cells directly. Owing to the mild cytotoxicity, E5 exhibited acceptable safety *in vivo*. Our previous work showed that the administration of E5 every other day in healthy BALB/c mice did not show detectable side effects, including unchanged organs weight, routine clinical parameters of serum, and no pathological changes in H&E staining of heart, kidney, lung and spleen.^22^

In addition, it should be noted that the antiangiogenic effects of CXCR4 antagonists both *in vitro* and *in vivo* are still elusive. Here, we report a new mechanism of E5 greatly decreasing tumor microenvironment angiogenesis by inhibiting the CXCL12-induced recruitment of endothelial progenitor cells in addition to its inhibitory effects on tumor cells. Our findings show that E5 inhibited the CXCL12-induced migration of vascular endothelial cells *in vitro* and reduced the formation of tumor vasculature, as evidenced by the downregulated levels of CD31 *in vivo*. This strongly indicates an additional role of E5 in the treatment of breast cancer by exerting antiangiogenic effects. In addition, E5 enhanced the anti-tumor efficacy of both CTX and PTX, even though the pharmacological mechanism of the two reagents are totally different. This was basically consistent with the previous result that E5 improved the efficacy of both vincristine and CTX in the treatment of leukemia.^21,22^ Collectively, these results strongly suggest that E5 could be a universal sensitizer to multiple chemotherapeutics. Furthermore, E5 has a half-life of 10 h, exhibiting high stability *in vivo*, and thus has sufficient potency to be administered less during cancer treatment. Because E5 can also interact with human serum albumin (HSA),^37^ circulating E5 together with HSA increased its blood circulation, which mostly likely contributed to its stability and anti-tumor effects.

In conclusion, E5 disrupted the interaction of 4T1 cells with stromal cells, which enhanced the sensitivity of the tumor cells to chemotherapeutics *in vitro* and *in vivo*. In addition, E5 inhibited tumor angiogenesis in breast cancer mice by suppressing the recruitment of endothelial cells. Therefore, E5 is expected to be a potential therapeutic agent to improve the clinical benefits of the current breast cancer therapies.
