# Supplementary material for: Targeting the CXCR4/CXCL12 axis with the peptide antagonist E5 to inhibit breast tumor progression
Source: Signal Transduct Target Ther. 2017 Aug 11;2:17033–. doi: 10.1038/sigtrans.2017.33 (PMC5661635; doi:10.1038/sigtrans.2017.33)
Supplement: Supplementary Figures [file sigtrans201733-s1.doc]

**Targeting CXCR4/CXCL12 axis by a peptide antagonist E5 to inhibit breast tumour progression**

Hua Guo^1*^, Yangyang Ge ^1*^, Xiaojin Li^1^, Yanlian Yang^2,3^, Jie Meng^1^, Jian Liu^1^, Chen Wang^2,3¶^, Haiyan Xu^1¶^

1. Institute of Basic Medical Sciences, Chinese Academy of Medical Sciences & Peking Union Medical College，Beijing 100005，P. R. China.
2. CAS Center for Excellence in Nanoscience，National Center for Nanoscience and Technology, Beijing 100190, P. R. China
3. University of Chinese Academy of Sciences, Beijing 100049, P. R. China

^*^ These authors contributed equally to this work

^¶^Corresponding authors: Haiyan Xu: [xuhy@pumc.edu.cn](mailto:xuhy@pumc.edu.cn) and Chen Wang: [wangch@nanoctr.cn](mailto:wangch@nanoctr.cn)

**Supporting Information**


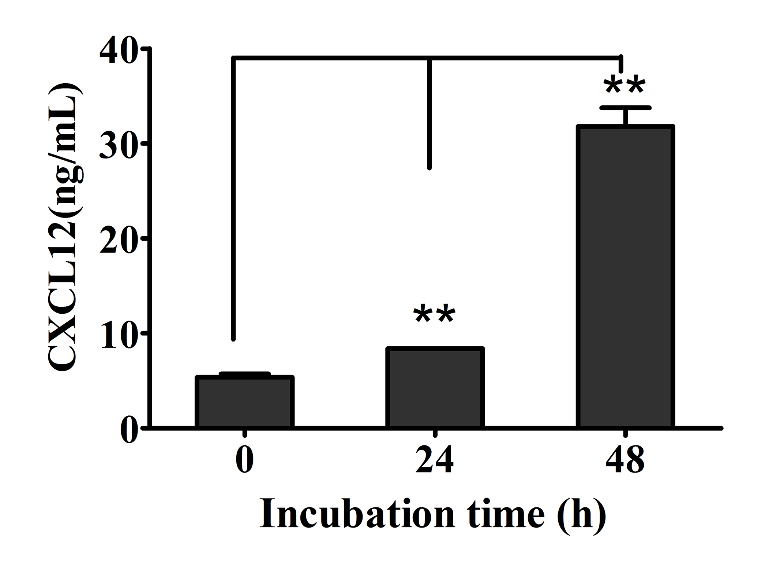


**Figure S1:**

The CXCL12 level secreted by MS-5 cell. MS-5 cells of 1ⅹ10^5^/well were seeded in 24-well plate with 800 mL medium and incubated for 24 h or 48 h at 37°C.The medium was collected and measured using Mouse CXCL12 Quantikine Elisa Kit.The data are presented as mean ± SD (n = 3). The * represents P < 0.05 and ** represents P < 0.01 respectively.


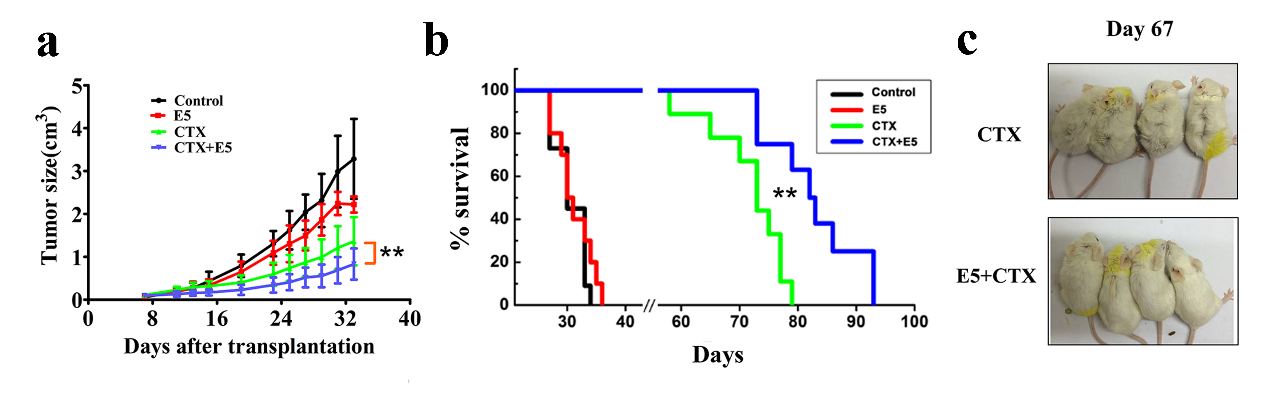


**Figure S2:**

Antitumor effect of E5 combination with CTX in breast cancer mice (n = 9). BALB/c mice were subcutaneously inoculated with 4T1 cells. After 7 days, mice in each group were subcutaneously injected with E5 every other day or intraperitoneal treated with CTX once a week. (a) The tumor growth of mice is depicted by tumor volume (mean ± SD). (b) The Survival of mice during the treatment. (c) The condition of mice in CTX group and combination treatment group on 67th. The ** represents P < 0.01.


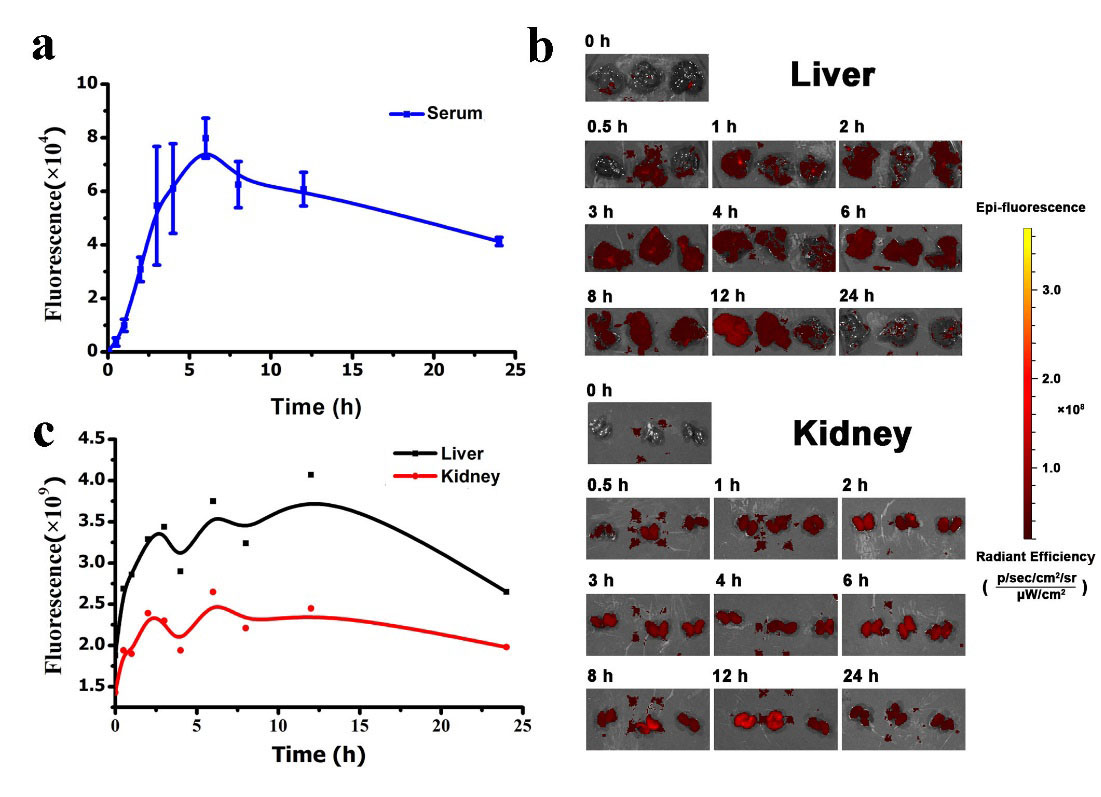


**Figure S3:**

Pharmacokinetic profile of free FITC after subcutaneous administration to mice at different time points (n = 3). (a) The fluorescent intensity in the serum samples after injection. (b) The fluorescent images of livers and kidneys at different time points. (c) The fluorescent intensity curve of liver and kidney.
